# A novel fluorescein sodium-based screening platform for the identification of sphingoid base-producing *Wickerhamomyces ciferrii* mutants

**DOI:** 10.3389/fbioe.2025.1548051

**Published:** 2025-02-26

**Authors:** Jun Su Kang, Seong-Rae Lee, Minju Lee, Eunha Kim, Pyung Cheon Lee

**Affiliations:** Department of Molecular Science and Technology and Advanced College of Bio-convergence Engineering, Ajou University, Suwon, Republic of Korea

**Keywords:** sphingolipids, fluorescein sodium, screening, *Wickerhamomyces ciferrii*, sphingoid bases, sphingosine

## Abstract

The efficient identification of microbial strains capable of producing rare sphingoid bases, such as sphingosine and sphinganine, is critical for advancing microbial fermentation processes and addressing increasing industrial demands. *Wickerhamomyces ciferrii*, a non-conventional yeast, naturally overproduces tetraacetyl phytosphingosine (TAPS); however, the production of other valuable sphingoid bases, including sphingosine, sphinganine, and triacetyl sphingosine, remains a key target. In this study, we developed a novel screening method utilizing fluorescein sodium, a selective fluorescent dye that specifically reacts with non-acetylated sphingoid bases—sphinganine, sphingosine, and phytosphingosine—while exhibiting no reactivity with TAPS. A mutant library of *W. ciferrii* was generated via gamma-ray mutagenesis and screened using fluorescence-activated cell sorting (FACS). Mutants exhibiting high fluorescence intensity, indicative of non-acetylated or partially acetylated sphingoid base production, were isolated through three rounds of sorting and further validated via HPLC analysis. This approach successfully identified three mutant strains: P41C3 (sphingosine-producing), M01_5 (sphinganine-producing), and P41E7 (triacetyl sphingosine-producing). Among them, the P41C3 mutant achieved a sphingosine titer of 36.7 mg/L during shake-flask cultivation, accompanied by a significant reduction in TAPS production, indicating a redirection of metabolic flux. This study demonstrates the utility of fluorescein sodium as a selective screening dye for sphingoid base-producing strains and establishes an effective platform for the metabolic engineering of *W. ciferrii* to enhance the production of industrially significant sphingolipids.

## Introduction

Lipids are essential cellular components, playing critical roles in maintaining structural integrity, energy storage, and signal transduction (Loix et al., 2023; Yoon et al., 2021). Among the diverse classes of lipids, sphingolipids are particularly noteworthy for their structural complexity and functional significance (Cowart and Obeid, 2007; Montefusco et al., 2014). These molecules share a common core structure, the sphingoid base, which consists of long-chain amino alcohols such as sphingosine, sphinganine, and phytosphingosine. Sphingoid bases serve as essential precursors for the synthesis of various sphingolipid derivatives, including ceramides, glycosphingolipids, and sphingomyelins (Zheng et al., 2006).

**FIGURE 1:**
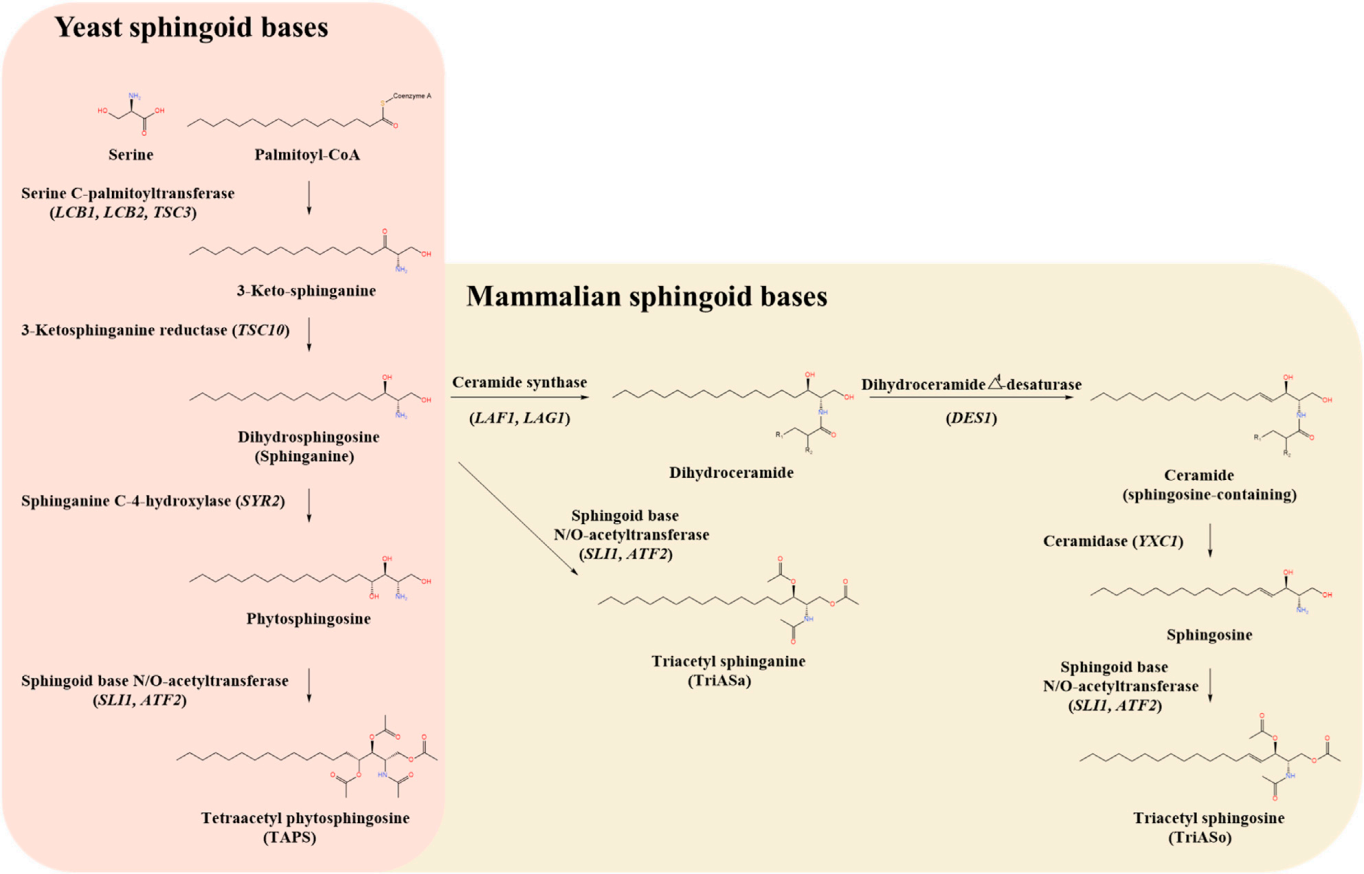
Comparative biosynthetic pathways of sphingoid bases in yeast and mammals. The schematic illustrates the biosynthetic pathways of sphingoid bases in yeast (*Wickerhamomyces ciferrii*) (left panel, pink background) and mammals (right panel, beige background), highlighting key intermediates, enzymes, and genes involved. The pathway begins with the condensation of serine and palmitoyl-CoA, catalyzed by serine C-palmitoyltransferase (LCB1, LCB2, TSC3), which represents the rate-limiting step in sphingoid base biosynthesis. The resulting 3-keto-sphinganine is subsequently reduced by 3-ketosphinganine reductase (TSC10) to form dihydrosphingosine (sphinganine). In yeast, sphinganine undergoes hydroxylation at the C-4 position by sphinganine C-4-hydroxylase (SYR2) to produce phytosphingosine. Phytosphingosine is then acetylated by sphingoid base N/O-acetyltransferases (SLI1, ATF2), leading to the formation of tetraacetyl phytosphingosine (TAPS), a major secreted sphingoid derivative in *W. ciferrii*. In mammals, sphinganine follows an alternative route, either being acetylated to form triacetyl sphinganine (TriASa) or converted into dihydroceramide via ceramide synthase (LAF1, LAG1). Dihydroceramide undergoes desaturation by dihydroceramide Δ4-desaturase (DES1) to produce ceramide, which can be further hydrolyzed by ceramidase (YXC1) to release sphingosine. Sphingosine can be subsequently acetylated by sphingoid base N/O-acetyltransferases (SLI1, ATF2) to generate triacetyl sphingosine (TriASo).

The biosynthesis of sphingoid bases involves a series of enzymatic reactions (Bartke and Hannun, 2009; Börgel et al., 2012). The initial step is the condensation of serine and palmitoyl-CoA, catalyzed by serine palmitoyltransferase (SPT), forming 3-keto-sphinganine. This intermediate is then reduced to sphinganine by 3-keto-sphinganine reductase (TSC10p). Sphinganine undergoes further modifications to generate diverse sphingoid bases. For example, phytosphingosine is synthesized via hydroxylation of sphinganine by sphinganine C-4-hydroxylase (SYR2p) and subsequently acetylated by acetyltransferases SLI1p and ATF2p to form tetraacetyl phytosphingosine (TAPS). Similarly, sphingosine is derived from sphinganine through sequential modifications by ceramide synthase (LAG1p/LAF1p), dihydroceramide Δ4-desaturase (DES1p), and ceramidase (YXC1p), with subsequent acetylation leading to formation of triacetyl sphingosine (TriASo). Alternatively, sphinganine can be directly acetylated to yield triacetyl sphinganine (TriASa) (Figure 1).

Sphingoid bases play critical roles in membrane structure and function, being present in cellular membranes as well as intracellular organelles such as the endoplasmic reticulum (ER) and the Golgi apparatus, where they contribute to sphingolipid metabolism (Futerman and Hannun, 2004). While the intracellular localization of biosynthesized triacetylated sphingoid bases, such as TriASo and TriASa, remains unclear, previous studies suggest that these hydrophobic molecules are likely retained within membranes or associated with intracellular lipid structures (Börgel et al., 2012). In contrast, TAPS, which is naturally produced by *Wickerhamomyces ciferrii*, has been reported to be secreted extracellularly (Maister et al., 1962), potentially through initial membrane association before export due to its strong hydrophobic nature.

Beyond their biological functions, sphingoid bases, particularly sphingosine and sphinganine, are valuable in the cosmetic and dermatological industries due to their skin-protective properties and ability to inhibit melanin synthesis (Coderch et al., 2003; Kim et al., 2002). Additionally, sphingosine has gained attention for its involvement in abnormal metabolism associated with various diseases, including cancer and Alzheimer’s disease. Recent studies have focused on developing advanced screening methods for sphingosine detection (He et al., 2010; Ogretmen and Hannun, 2004), including fluorescence-based assay using near-infrared fluorescent probes such as DMS-X (X = 2F, F, Cl, Br and I) and BODIPY FL dye, as well as enzymatic approaches. For example, *Escherichia coli* diacylglycerol kinase has been utilized to phosphorylate modified sphingosine derivatives, such as N-hexanoyl-sphingosine, incorporating a radioactive phosphate from ^32^P-labeled ATP, to achieve highly sensitive detection at picomole-to-nanomole levels (Chen et al., 2024; Rudd et al., 2020; Van Veldhoven et al., 1989). While these methods have significantly improved sphingosine detection in living cells, their application in screening sphingosine-producing yeast strains remains limited.

The increasing demand for sphingoid bases has driven efforts to develop microbial production systems as an alternative to plant- or animal-derived sources. Microbial production offers advantages such as scalability, cost-effectiveness, and sustainability (Biermann et al., 2011; Lennen and Pfleger, 2013). Both metabolic engineering (Bae et al., 2003; Kim et al., 2010; Börgel et al., 2012; Han et al., 2021; Jang et al., 2024; Schorsch et al., 2009; Schorsch et al., 2012; Yoo et al., 2023) and random mutagenesis (Choi et al., 2021) have been employed to enhance microbial sphingoid base production by leveraging microbial systems’ versatility to achieve higher yields and tailored biosynthesis.

Yeasts, particularly *Wickerhamomyces ciferrii*, have emerged as promising hosts for sphingoid base production due to their rapid growth, safety, and metabolic flexibility. *W. ciferrii* is particularly notable for its natural ability to overproduce and secrete TAPS, a commercially valuable sphingolipid derivative. Considerable progress has been made in optimizing TAPS production through strain improvement strategies (Choi et al., 2021; Eun and Lee, 2023; Yoo et al., 2023). Additionally, metabolic engineering approaches have explored to produced sphingosine-related compounds by introducing heterologous genes encoding sphingoid base biosynthetic enzymes into haploid strains (Börgel et al., 2012). However, targeted genome editing in diploid *W. ciferrii* remains challenging due to the strain’s preference for non-homologous end joining (NHEJ) over homologous recombination (HR), high genomic heterogeneity, and the absence of a complete and fully-phased diploid genome. These challenges necessitate alternative approaches, such as random mutagenesis, for strain improvement.

A mutant screening strategy utilizing syringomycin E, an antifungal phytotoxin secreted by *Pseudomonas syringae*, has previously been investigated as a selection method (Börgel et al., 2012). The *SYR2* gene is essential for yeast growth inhibition by syringomycin E, and mutations in *SYR2* confer resistance to this toxin (Cliften et al., 1996). Screening for *SYR2* mutants through spontaneous mutation followed by syringomycin E selection has been demonstrated (Börgel et al., 2012). However, the broader application of this approach is constrained by the limited commercial availability of syringomycin E. Consequently, efficient random mutagenesis strategies combined with robust screening methodologies are necessary for the identification of sphingoid base-overproducing mutant.

High-throughput screening systems employing fluorescent dyes, such as BODIPY, Nile Red, and Nile Blue, have been widely used for lipid detection (Kimura et al., 2004; Park et al., 2022; Vijayalakshmi et al., 2003). Among these, fluorescein sodium (uranine) is particularly notable for its low toxicity, cost-effectiveness, and high sensitivity at minimal concentrations, despite certain limitations such as photochemical instability and pH sensitivity (Weidner et al., 2011; Ken-Ichiro O’goshi and Serup, 2006). These attributes have facilitated its application in various scientific fields, including neurosurgery, ophthalmology, biological imaging, fluorescence resonance energy transfer (FRET) studies, and polymer research (Ahmed et al., 2001; Maurice, 1967; Patil and Patil, 2019; Yin et al., 2014; Zhao et al., 2019).

**FIGURE 2:**
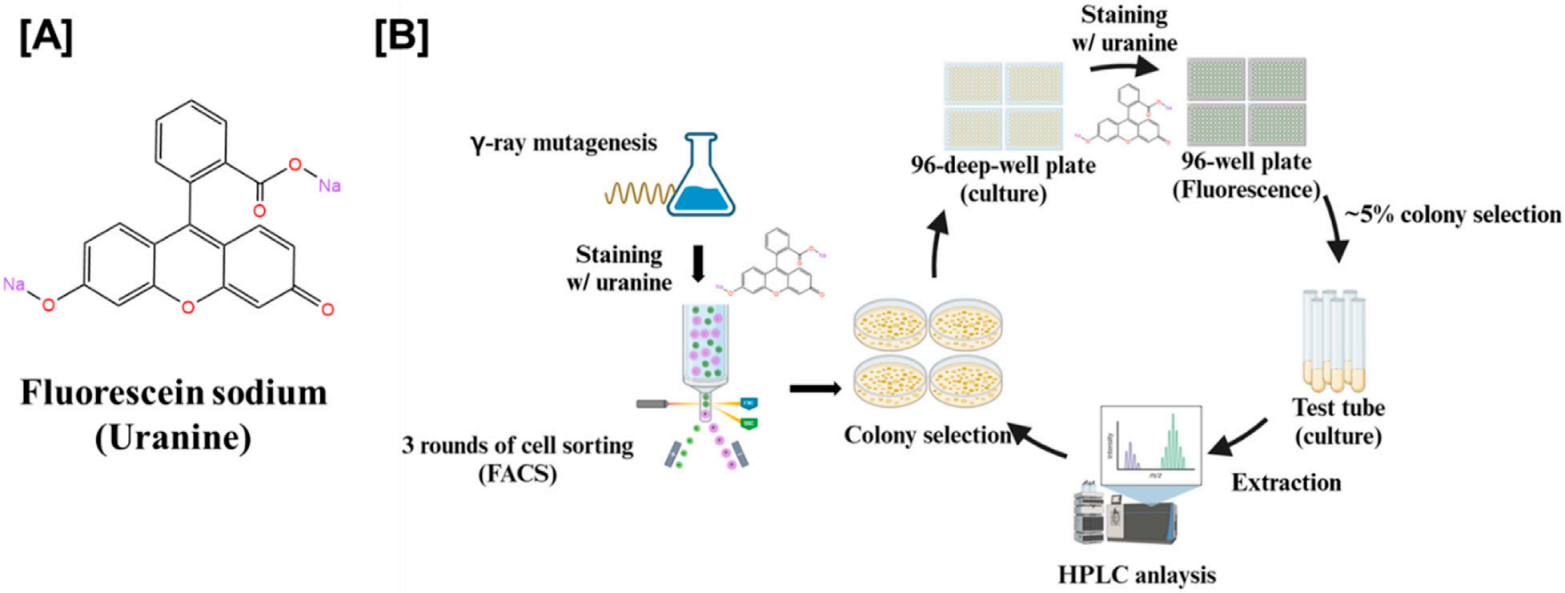
Screening workflow for sphingoid base-overproducing *W. ciferrii* mutants using fluorescein sodium staining and fluorescence-activated cell sorting (FACS). **(A)** Chemical structure of fluorescein sodium, a fluorescent dye used to stain yeast cells for assessing sphingoid base production. **(B)** Schematic representation of the mutant screening strategy. *W. ciferrii* cells were subjected to gamma-ray mutagenesis to induce genetic diversity. Mutagenized cells were stained with fluorescein sodium and sorted via FACS through three consecutive rounds to enrich for high-fluorescence populations, indicative of elevated sphingoid base production. The enriched mutants were cultured in 96-deep-well plates, followed by fluorescence-based selection to identify the top ∼5% of high-expressing colonies. The selected candidates were subsequently cultured in small-scale test tubes, and sphingoid base extraction was performed. Finally, high-performance liquid chromatography (HPLC) analysis was conducted to confirm and quantify sphingoid base production.

Despite its widely use, the mechanism of fluorescein sodium’s interaction with cellular components remains incompletely understood. The dye is known to penetrate cells, accumulate intracellularly, and bind to macromolecules (Braginskaja et al., 1993; Lietard et al., 2022). Notably, fluorescein sodium has been reported to interact with sphingosine, a key sphingoid base (Saito, 1960). Based on this property, we developed a fluorescein sodium-based screening method (Figure 2) to identify sphingoid bases-overproducing mutant from a *W. ciferrii* mutant library generated by gamma-ray mutagenesis (Choi et al., 2021). Given that TAPS biosynthesis shares an abundant precursor pool with sphingoid bases, we hypothesized that targeting *W. ciferrii* would facilitate the efficient identification of mutants with enhanced sphingoid bases production.

## Material and methods

### Strains, mutant library generation, and cultivation conditions

The diploid wild-type *Wickerhamomyces ciferrii* strain (F-60-10A, NRRL1031) was utilized for mutant library construction via gamma-ray mutagenesis, as previously described (Choi et al., 2021). Exponentially growing cultures (OD_600_ = 1.0) were exposed to a radiation dose of 0.3 kGy using a^60^Co γ-irradiator (IR-79, Nordion International Ltd., Ontario, Canada). Following irradiation, cells were preserved at −80°C with 20% (v/v) glycerol. For routine cultivation, single colonies of both wild-type and mutant strains were grown in YPD medium (10 g/L yeast extract, 20 g/L peptone, 20 g/L glucose) or YMglSC medium (2 g/L yeast extract, 2 g/L malt extract, 7 g/L peptone, 20 g/L glycerol, 10 mM CaCl_2_, 5 g/L serine) under aerobic conditions. Cell growth was monitored by the optical density measurement at 600 nm (OD_600_) using a SPECTRAmax PLUS384 spectrophotometer. For staining optimization, cells were cultivated in 2 mL YPD medium at 25°C with shaking at 250 rpm for 12 h. High-throughput screening was conducted in 96-deep-well plates containing 1 mL of YMglSC medium per well, incubated at 25°C with shaking at 800 rpm for 4 days. Mutant candidates exhibiting elevated fluorescence were further cultured in small scale test tubes containing 4 mL of YMglSC medium at 25°C with shaking at 250 rpm for 4 days. For shake-flask cultivation, mutant strains were pre-cultured in 2 mL of YPD medium for 12 h and subsequently transferred to 250 mL baffled flasks containing 50 mL of YMglSC medium, incubated at 25°C with shaking at 250 rpm for 4 days.

### Chemical synthesis of acetylated sphingoid bases

Triacetylated sphinganine and triacetylated sphingosine were synthesized via acetylation of sphinganine and sphingosine, respectively. In brief, 10 mg of sphinganine or sphingosine was reacted with excess acetic anhydride in pyridine as a solvent. The reaction progression was monitored by thin-layer chromatography (TLC) on silica gel 60 (F_254_ 0.25 mm) and visualized using phosphomolybdic acid (PMA) or ninhydrin staining, followed by heating. After 6 h, consumption of the starting materials and formation of the target compounds were confirmed. The reaction mixtures were extracted with ethyl acetate (EtOAc), washed with deionized water to remove residual pyridine, and dried over anhydrous sodium sulfate. The crude products were purified by flash liquid column chromatography (Biotage), yielding 14.1 mg of triacetyl sphinganine and 4.5 mg of triacetyl sphingosine as white solids. Structural elucidation was performed using ^1^H-nuclear magnetic resonance (NMR) spectroscopy on a JEOL 600 MHz spectrometer, while high-resolution mass spectrometry was conducted on an LTQ Orbitrap XL mass spectrometer equipped with an electrospray ionization (ESI) source (details in Supplementary Data 1).

### Fluorescence reactivity assay between fluorescein sodium and sphingoid bases

To evaluated the interaction of sphingoid bases with fluorescein sodium, solutions of sphinganine, sphingosine, phytosphingosine, tetraacetyl phytosphingosine (Avanti Polar Lipids), and the synthesized triacetyl sphinganine and triacetyl sphingosine were prepared at concentrations ranging from 1.9 to 1,000 μg/mL in ethanol. A 90 μL aliquot of each solution was mixed with 10 μL of fluorescein sodium (100 μg/L in deionized water), and fluorescence intensity was measured using a Cytation 3 plate reader (BioTek) in a 96-well format. Given the spectral proximity of fluorescein’s absorption (∼498 nm) and emission (∼517 nm) maxima, optical interference was observed. To mitigate spectral overlap, an optimized excitation wavelength of 475 nm and an emission wavelength of 515 nm were utilized (AAT Bioquest, Inc., 2025).

### Fluorescence-activated cell sorting (FACS) analysis

A 1 mL aliquot of wild-type *W. ciferrii* cells grown to OD_600_ = 10 was harvested by centrifugation and washed twice with 1 mL of phosphate-buffered saline (PBS, pH 7.4). The cell pellet was resuspended in fluorescein sodium solution (10 g/L) to achieve final staining concentrations of 0.1, 0.125, 0.25, 0.5, and 1 g/L, followed by incubation in the dark at room temperature for 1, 30, or 120 min. After staining, cells were washed three times with 1 mL of PBS to remove unbound dyes and subjected to fluorescence-activated cell sorting (FACS) using a BD Biosciences system equipped with a FITC channel (excitation 495 nm, emission 519 nm). The flow rate was maintained below 2,000 events per second to minimize signal variability, and approximately 100,000 cells were selectively enriched based on fluorescence intensity.

### Confocal microscopy imaging

A 1 mL aliquot of *W. ciferrii* cells were pelleted by centrifugation and washed twice with 1 mL of PBS, stained with fluorescein sodium, and subsequently washed to remove excess dye. Nuclear counterstaining was performed using 4′,6-diamidino-2-phenylindole (DAPI) at a final concentration of 300 nM for 1 min, followed by 1 mL of PBS wash. Imaging was conducted using a STELLARIS 5 Cryo confocal microscope (Leica), employing excitation wavelength of 405 nm for DAPI and 488 nm for fluorescein sodium, with appropriate emission filters.

### Extraction and quantification of sphingoid bases

Sphingoid base extraction was performed using a modified protocol based on Börgel et al. (2012) and Xie et al. (2014). Briefly, cultures were subjected to high-pressure heat treatment at 121°C for 5 min to lyse cells. After cooling, chloroform was added at twice the culture volume, and samples were vortexed vigorously for 15 min. Phase separation was achieved by centrifugation at 2,000 rpm for 10 min, and the lower chloroform layer was collected. The solvent was evaporated using an EZ-2 rotary evaporator (Genevac), and dried residues were resuspended in 1 mL of ethanol, followed by sonication at 40°C for 15 min.

High-performance liquid chromatography (HPLC) analysis was performed using an Agilent 1,260 series HPLC system equipped with a diode array detector and a ZORBAX Eclipse XDB-C18 column (150 mm × 4.6 mm, 5 μm, Agilent Technologies) at 20°C. The mobile phase consisted of 100% acetonitrile (solvent A) and 100% deionized water (solvent B). Gradient elution was carried out at a flow rate of 1 mL/min as follows: 45% solvent A at 0 min, increased to 80% at 18 min, 90% at 20 min, and 100% at 23 min, maintained at 100% until 25 min, then decreased to 45% at 28 min, and held at 45% until 30 min. Acetylated sphingoid bases were detected at 200 nm, while non-acetylated sphingoid bases (sphinganine, sphingosine, and phytosphingosine) were derivatized with an equal volume of *o*-phthalaldehyde (OPA) for 2 min and detected at 340 nm. Quantification was performed using calibration curves generated from authentic standards.

## Results

### Selective reactivity of fluorescein sodium with sphingoid bases and its effect on cell viability

**FIGURE 3:**
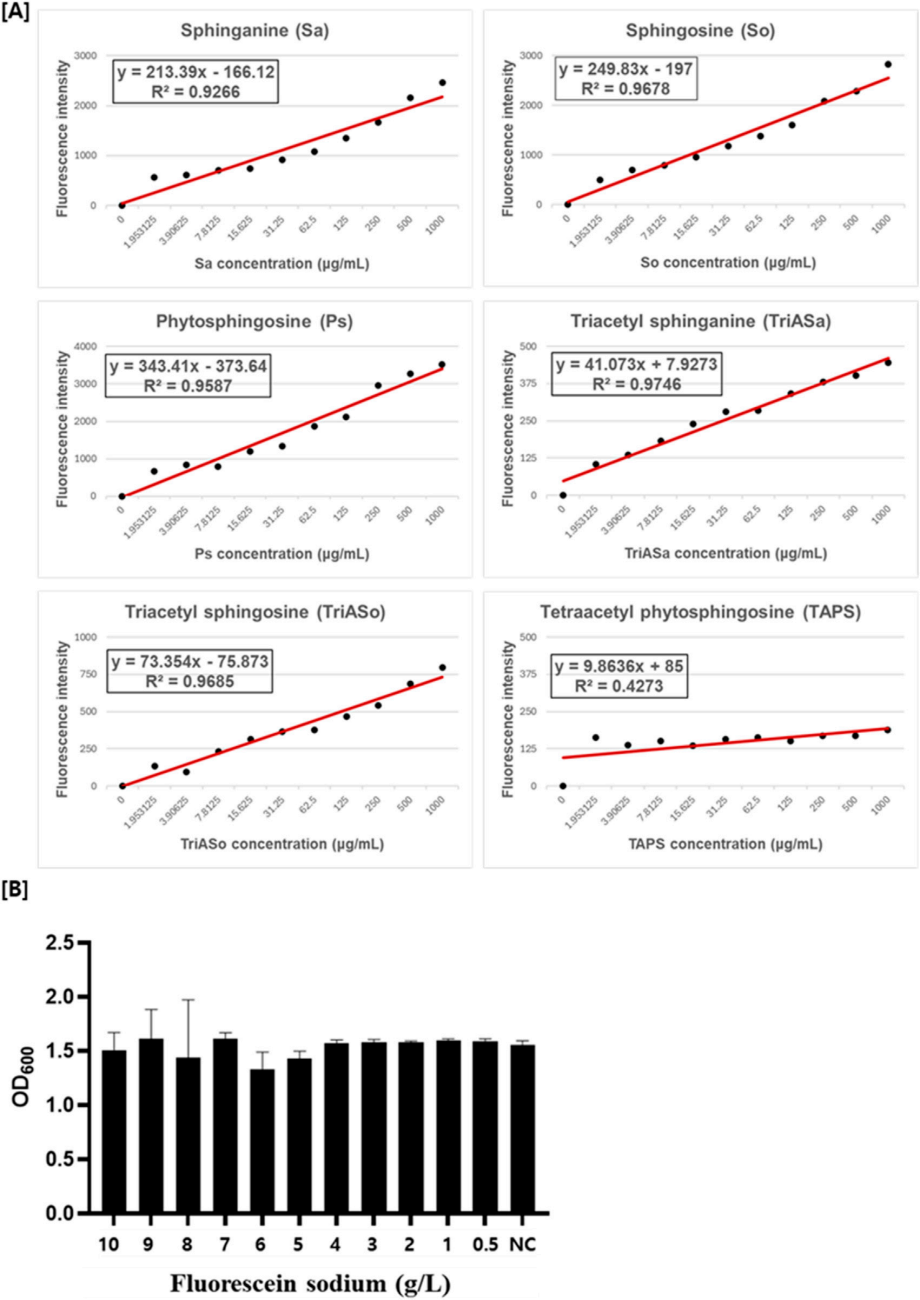
Fluorescein sodium reactivity with sphingoid bases and its effect on *W. ciferrii* viability. **(A)** Fluorescence intensity of fluorescein sodium in the presence of various sphingoid bases, including sphinganine (Sa), sphingosine (So), phytosphingosine (Ps), triacetyl sphinganine (TriASa), triacetyl sphingosine (TriASo), and tetraacetyl phytosphingosine (TAPS). Fluorescence was measured at an excitation wavelength of 476 nm and an emission wavelength of 515 nm using a black-walled 96-well plate. Non-acetylated (Sa, So, Ps) and acetylated (TriASa, TriASo) sphingoid bases exhibited a concentration-dependent increase in fluorescence intensity, whereas TAPS showed minimal fluorescence, indicating weak reactivity with fluorescein sodium. Linear regression equations and R^2^ values are displayed for each sphingoid base. **(B)** Cytotoxicity assessment of fluorescein sodium in wild-type *W. ciferrii*. Cells were cultured in YPD medium supplemented with fluorescein sodium at concentrations ranging from 0.5 to 10 g/L, and cell growth was monitored to evaluate potential inhibitory effects. No significant growth inhibition was observed across all tested concentrations. “NC” represents the negative control, cultured in YPD medium without fluorescein sodium. Error bars indicate the standard deviation (SD) of three independent biological replicates (n = 3), each measured in technical triplicates.

To evaluate the suitability of fluorescein sodium as a selective screening dye for sphingoid base-producing strains, its reactivity was assessed with five distinct sphingoid bases: sphinganine, sphingosine, phytosphingosine, triacetyl sphinganine (synthesized in this study; Supplementary Data 1), and triacetyl sphingosine (synthesized in this study; Supplementary Data 1) as well as TAPS. Fluorescence measurements revealed a concentration-dependent increase in fluorescence intensity for the three non-acetylated sphingoid bases (sphinganine, sphingosine, and phytosphingosine) upon interaction with fluorescein sodium (Figure 3A). Similarly, the acetylated sphingoid bases, triacetyl sphinganine and triacetyl sphingosine, exhibited proportional fluorescence responses, indicating their ability to react with fluorescein sodium. In contrast, TAPS, the major sphingolipid derivate produced by *W. ciferrii*, displayed negligible fluorescence under the identical conditions. These findings confirm the selective reactivity of fluorescein sodium with sphingoid bases while remaining unresponsive to TAPS, thus enabling the differentiation of mutant strains producing elevated levels of sphingoid bases.

To determine the potential cytotoxicity of fluorescein sodium, *W. ciferrii* cells were cultured in the presence of fluorescein sodium at concentrations ranging from 0 to 10 g/L. Optical density (OD_600_) measurement showed no significant differences in cell growth across all tested concentration (Figure 3B), indicating that fluorescein sodium dose not adversely affect *W. ciferrii* viability. These results demonstrate that fluorescein sodium is a non-toxic and effective fluorescent probe for live-cell screening of sphingoid base-producing strains.

### Optimization of fluorescein sodium staining for FACS analysis

**FIGURE 4:**
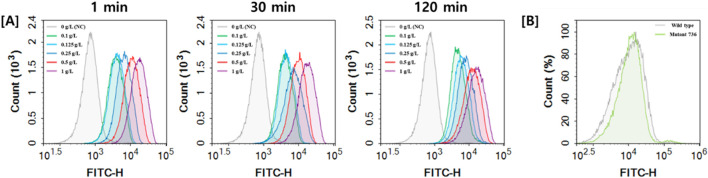
Optimization of fluorescein sodium staining conditions and *in vivo* validation of its specificity. **(A)** FACS analysis of wild-type *W. ciferrii* cells stained with fluorescein sodium at concentrations of 0 g/L (gray), 0.1 g/L (green), 0.125 g/L (light blue), 0.25 g/L (blue), 0.5 g/L (red), and 1 g/L (purple) after incubation for 1, 30, and 120 min. Fluorescence intensity was measured to determine the optimal staining conditions by assessing the effects of dye concentration and incubation time on fluorescence signal distribution. **(B)** FACS analysis comparing the fluorescence profiles of wild-type *W. ciferrii* (gray) and the TAPS-overproducing mutant strain 736 (green) after fluorescein sodium staining. No significant difference in fluorescence intensity was observed between the two strains, indicating that fluorescein sodium does not interreact with TAPS *in vivo*. This confirms that the dye specifically reacts with non-acetylated sphingoid bases while remaining unresponsive to TAPS in living *W. ciferrii* cells.

To facilitate fluorescence-activated cell sorting (FACS) for identifying mutant strains overproducing sphingoid bases, optimal staining conditions using fluorescein sodium were established. Live *W*. *ciferrii* cells (grown to OD_600_ = 10) were treated with varying concentrations of fluorescein sodium (0.1–1 g/L) and incubated for different times (1, 30, and 120 min). Fluorescence intensity increased with higher dye concentrations, with 1 g/L fluorescein sodium yielding the most distinct peak shift in FACS analysis (Figure 4A). Incubation time had minimal effect on fluorescence intensity, with a 1-min staining period proving sufficient for effective and reproducible staining. These optimized conditions were selected for all subsequent FACS experiments. To validate the *in vivo* specificity of fluorescein sodium for TAPS, wild-type *W. ciferrii* and a TAPS-overproducing mutant strain (736 strain; Choi et al., 2021) were stained under these optimized conditions and analyzed by FACS. Both strains exhibited similar fluorescence intensities, indicating that fluorescein sodium does not significantly react with intracellular TAPS. This finding confirms the utility of fluorescein sodium as a selective dye for screening mutant strains that produce non-acetylated or partially acetylated sphingoid bases, rather than TAPS.

### Fluorescein sodium-based screening of randomly mutagenized *W*. *ciferrii* strains

To isolate *W. ciferrii* mutants with enhanced sphingoid base production from a mutant library of *W*. *ciferrii* generated through gamma-ray mutagenesis, fluorescence-activated cell sorting (FACS) was performed using fluorescein sodium as a selective staining reagent. Mutant cells grown to OD_600_ = 10 in YPD medium were stained with fluorescein sodium (1 g/L) and sorted based on fluorescence intensity. Cells exhibiting elevated fluorescence were selectively collected from gates P2, P3, and P4 during the primary sorting process (Supplementary Data 2). Subsequent secondary and tertiary rounds of sorting were performed to refine the mutant selection.

Following FACS, enriched cell populations were streaked onto YPD agar plates, and 100 individual colonies were cultivated in a 96-deep-well plate containing 1 mL of YMglSC medium per well at 25°C with shaking at 800 rpm for 4 days. Fluorescence intensity was normalized to cell growth (OD_600_), and the top 5% of colonies exhibiting the highest fluorescence-to-growth ratios were selected for further evaluation. These candidate mutants were subsequently cultured in 4 mL of YMglSC medium, and their sphingoid base production was assessed using high-performance liquid chromatography (HPLC).

**FIGURE 5:**
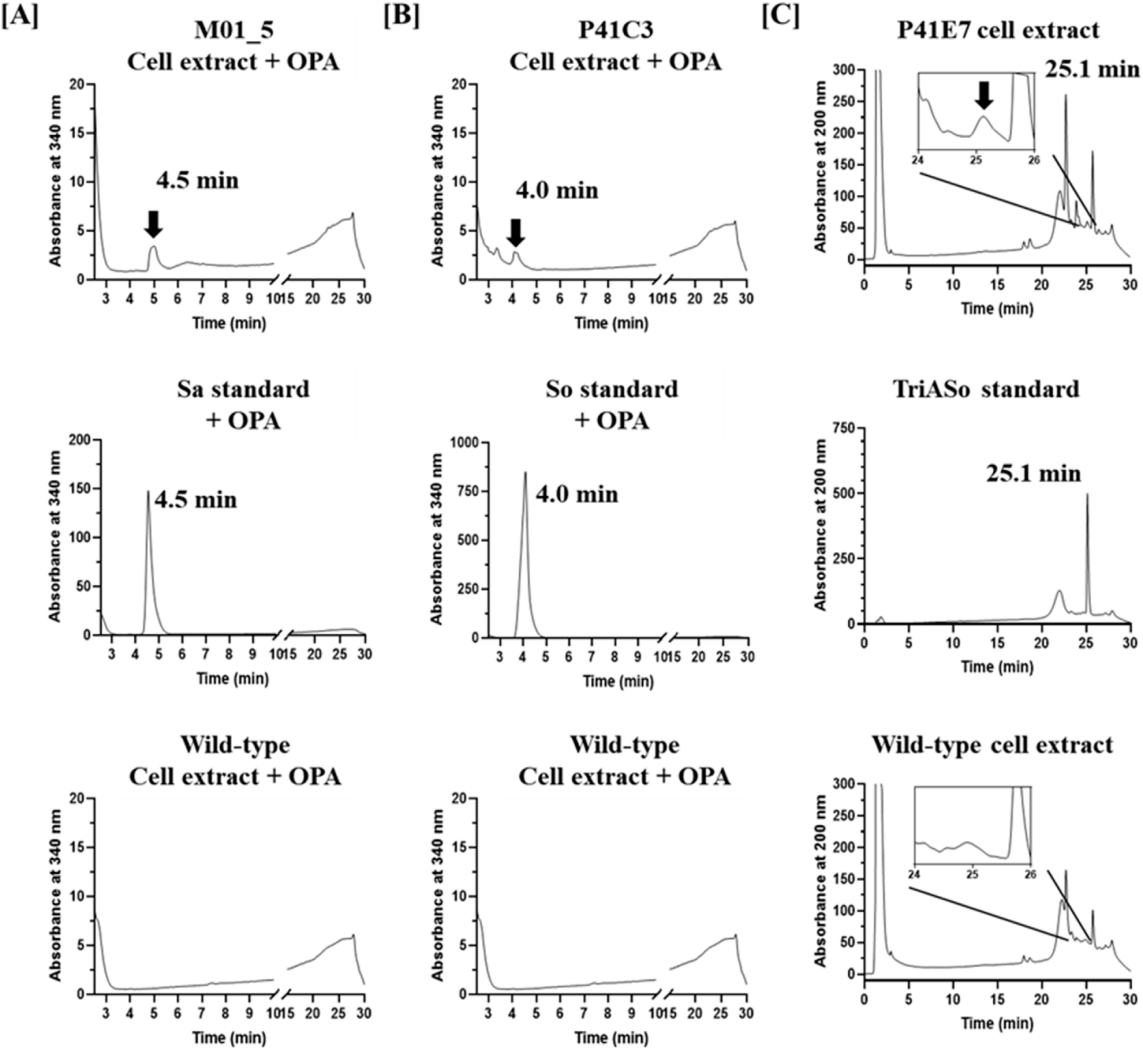
HPLC analysis of sphingoid base production in *W. ciferrii* mutants isolated through fluorescein sodium-based screening. **(A)** HPLC chromatograms of the sphinganine-overproducing mutant M01_5 (upper), sphinganine (Sa) standard (middle), and wild-type *W. ciferrii* extract (lower). Samples were derivatized with *o*-phthalaldehyde (OPA) and detected at 340 nm. The presence of a distinct peak at the retention time of the Sa standard in the M01_5 extract confirms enhanced sphinganine production. **(B)** HPLC chromatograms of the sphingosine-overproducing mutant P41C3 (upper), sphingosine (So) standard (middle), and wild-type *W. ciferrii* extract (lower), analyzed at 340 nm after OPA derivatization. The alignment of a major peak in the P41C3 extract with the So standard indicates increased sphingosine production in this mutant. **(C)** HPLC chromatograms of the triacetyl sphingosine-overproducing mutant P41E7 (upper), triacetyl sphingosine (TriASo) standard (middle), and wild-type *W. ciferrii* extract (lower), detected at 200 nm without OPA derivatization. The appearance of a peak corresponding to the TriASo standard in the P41E7 extract confirms the accumulation of triacetyl sphingosine in this mutant.

HPLC analysis revealed distinct production profiles among the selected mutants. The M01_5 mutant strain exhibited a major peak corresponding to sphinganine, confirmed by co-elution with the sphinganine (Sa) standard after *o*-phthalaldehyde (OPA) derivatization (Figure 5A), indicating its classification as a sphinganine-overproducing strain. The P41C3 strain showed a peak corresponding to sphingosine, which aligned with the sphingosine (So) standard following OPA derivatization (Figure 5B), confirming its identity as a sphingosine-overproducing strain. In contrast, the P41E7 mutant strain displayed a distinct peak at the retention time corresponding to triacetyl sphingosine, consistent with the TriASo standard, identifying it as a triacetyl sphingosine-overproducing strain (Figure 5C). Notably, wild-type *W. ciferrii* cell extracts exhibited no detectable levels of sphinganine, sphingosine, or triacetyl sphingosine, underscoring the metabolic differences between the wild-type and mutant strains. These findings demonstrate the effectiveness of fluorescein sodium-based FACS screening for the rapid and selective isolation of *W. ciferrii* mutants with enhanced sphingoid base production. The approach successfully differentiated mutants based on intracellular sphingoid base accumulation, allowing targeted selection of strains with distinct metabolic profiles.

### Sphingoid base production in isolated mutant strains

The sphingoid base production profiles of the isolated *W. ciferrii* mutants were further characterized in shake-flask cultures to assess their biosynthetic capacity. The sphingosine-overproducing strain P41C3 exhibited a significantly enhanced sphingosine titer of 36.7 mg/L, accompanied by a secondary production of 3.64 mg/L sphinganine. Notably, the production of tetraacetyl phytosphingosine (TAPS) in P41C3 was markedly reduced to 51.3 mg/L, representing a 41.2% decrease compared to the wild-type strain. This substantial reduction in TAPS levels suggests a successful metabolic shift favoring sphingosine biosynthesis at the expense of TAPS production, highlighting the strain’s potential for targeted metabolic engineering. Similarly, the sphinganine-overproducing strain M01_5 demonstrated a sphinganine titer of 12.0 mg/L, confirming its capacity for enhanced production of this precursor molecule. The triacetyl sphingosine-overproducing strain P41E7 accumulated 17.5 mg/L of triacetyl sphingosine, further validating the metabolic versatility of the selected mutants and their potential for biotechnological applications in sphingolipid production.

To further validate the specificity of fluorescein sodium in selectively detecting non-acetylated sphingoid bases *in vivo*, fluorescence-activated cell sorting (FACS) and confocal microscopy were performed on wild-type *W. ciferrii*, the TAPS-overproducing mutant 736 strain, and the sphingosine-overproducing mutant P41C3 strain. The P41C3 mutant exhibited a markedly higher fluorescence signal compared to both the wild-type and 736 strains (Supplementary Data 3), consistent with its increased intracellular sphingosine levels. These findings corroborate the selective interaction between fluorescein sodium and non-acetylated sphingoid bases, further reinforcing the efficacy of this fluorescence-based screening approach. Collectively, these results demonstrate the successful application of fluorescein sodium-based FACS screening in isolating *W. ciferrii* mutants with enhanced sphingoid base production. The identified strains exhibit significant potential for industrial applications, offering a sustainable microbial platform for the biosynthesis of high-value sphingoid bases.

## Discussion

This study presents a novel fluorescence-based screening strategy utilizing fluorescein sodium for the identification of *W. ciferrii* mutants with enhanced sphingoid base production. By exploiting the selective interaction between fluorescein sodium and non-acetylated sphingoid bases, this approach overcomes key limitations associated with conventional screening methods and provides an efficient platform for strain improvement.

Unlike traditional lipid-staining dyes such as Nile Red and BODIPY, fluorescein sodium exhibited specific reactivity toward sphinganine, sphingosine, and phytosphingosine, while showing negligible interaction with TAPS. This specificity facilitated the selective enrichment of mutant strains that redirect metabolic flux from TAPS biosynthesis toward the production of rare sphingoid bases. The differential reactivity between acetylated and non-acetylated sphingoid bases suggests that fluorescein sodium selectively interacts with free amino groups in non-acetylated sphingoid bases, a hypothesis supported by a previous report of fluorescein complex formation with sphingosine (Saito, 1960). Conversely, the minimal fluorescence observed in fully acetylated sphingoid bases, including TAPS, likely results from steric hindrance or enhanced chemical stability that inhibits such interactions.

The application of this screening platform led to the successful isolation of three distinct *W. ciferrii* mutant strains: P41C3 (sphingosine-producing), M01_5 (sphinganine-producing), and P41E7 (triacetyl sphingosine-producing). Among them, P41C3 exhibited the highest sphingosine titer (36.7 mg/L), accompanied by a significant reduction (41.2%) in TAPS production, indicating a shift in metabolic flux. These findings demonstrate the utility of fluorescein sodium-based FACS screening in rapidly identifying mutants with altered sphingoid base metabolism, enabling the discovery of strains suitable for industrial-scale production.

Despite the effectiveness of this approach, certain limitations necessitate further optimization or investigation. First, the precise mechanism underlying fluorescein sodium’s selective interaction with sphingoid bases remains incompletely understood. While our findings suggest that fluorescein sodium interacts with free amino groups in non-acetylated sphingoid bases, the fluorescence response of partially acetylated sphingoid bases, such as mono- and di-acetylated sphingoid bases, remains unclear. Additionally, triacetyl sphinganine and triacetyl sphingosine may undergo partial deacetylation under physiological conditions, potentially generating transient fluorescence signals. Further biochemical investigations, including structural characterization and kinetic analyses, are required to confirm these hypotheses and elucidate the molecular basis of fluorescein sodium specificity.

Second, the efficiency of fluorescein sodium-based screening was relatively modest, yielding only three confirmed high-producing mutants from the mutant library. This suggests that either the screening parameters require further optimization or that fluorescein sodium may not effectively detect all sphingoid base variants. Refinements such as optimizing *in vivo* dye concentration, incubation time, and FACS gating strategies, could enhance the screening resolution and improve recovery rate of target-producing strains. Additionally, integrating complementary analytical techniques, such as mass spectrometry-based metabolomics, could provide a more comprehensive evaluation of intracellular sphingoid base accumulation.

Another key finding of this study is the metabolic competition between TAPS and sphingoid base biosynthesis in *W. ciferrii*. The significant reduction in TAPS production in the P41C3 mutant suggests that restricting the metabolic flux toward TAPS could be an effective strategy for enhancing sphingosine production. Given that TAPS biosynthesis represents a major metabolic sink in *W. ciferrii*, future metabolic engineering strategies could focus on downregulating or knocking out key enzymes involved in TAPS biosynthesis to further increase sphingoid base yields.

In conclusion, this study demonstrates the feasibility of fluorescein sodium-based screening for the rapid identification of *W. ciferrii* mutants with enhanced sphingoid base production. By selectively differentiating between non-acetylated and acetylated sphingoid bases, this high-throughput platform enables efficient mutant selection. The successful redirection of metabolic flux from TAPS toward sphingosine production underscores the potential of *W. ciferrii* as a microbial chassis for industrial sphingoid base production. By integrating synthetic biology tools such as CRISPR-based pathway engineering, this platform can drive development of sustainable microbial production systems for high-value sphingolipid in pharmaceutical, cosmetic, and biomedical applications.

## Data Availability

The original contributions presented in the study are included in the article/Supplementary Material, further inquiries can be directed to the corresponding author.
