# Supplementary material for: A novel fluorescein sodium-based screening platform for the identification of sphingoid base-producing Wickerhamomyces ciferrii mutants
Source: Front Bioeng Biotechnol. 2025 Feb 26;13:1548051. doi: 10.3389/fbioe.2025.1548051 (PMC11897276; doi:10.3389/fbioe.2025.1548051)
Supplement: Supplementary file 1 [file DataSheet1.docx]

**Supplementary data 1**

**Chemical synthesis of triacetyl sphinganine / triacetyl sphingosine**

**(2R,3S)-2-Acetamidooctadecane-1,3-diyl diacetate (1) – Triacetyl sphinganine**

Acetic anhydride (47 µL, 0.498 mmol, 15 equiv.) was added to a solution of sphinganine (10 mg, 0.033 mmol, 1 equiv.) and DMAP (0.4 mg, 0.003 mmol, 0.1 equiv.) in pyridine (1 mL). The reaction mixture was stirred for 6 hours at room temperature. After reaction completion, the reaction mixture was extracted to EtOAc and washed with DW. The organic layer was dried over sodium sulfate. The residue was purified by flash column chromatography (1:99 to 50:50; EtOAc/Hexane) to give a white solid (14.1 mg, 99%).^1^H NMR (600 MHz, CDCl_3_) δ 5.89 (d, *J* = 9.1 Hz, 1H), 4.91 (dt, *J* = 8.1, 5.2 Hz, 1H), 4.39 (m, *J* = 9.3, 5.7, 3.9 Hz, 1H), 4.25 (dd, *J* = 11.6, 6.1 Hz, 1H), 4.06 (dd, *J* = 11.6, 3.9 Hz, 1H), 2.07 (d, *J* = 7.2 Hz, 6H), 2.00 (s, 3H), 1.60 (tt, *J* = 8.9, 4.6 Hz, 2H), 1.25 (d, *J* = 4.5 Hz, 26H), 0.88 (t, *J* = 7.0 Hz, 3H). HRMS (ESI): [M+H]^+^ m/z calcd. 428.33705 for C_24_H_45_NO_5_^+^, found 428.33704

**(2R,3S,E)-2-Acetamidooctadec-4-ene-1,3-diyl diacetate (2) – Triacetyl sphingosine**

Acetic anhydride (32 µL, 0.334 mmol, 10 equiv.) was added to a solution of sphingosine (10 mg, 0.033 mmol, 1 equiv.) and DMAP (0.4 mg, 0.003 mmol, 0.1 equiv.) in pyridine (1 mL). The reaction mixture was stirred for 6 hours at room temperature. After reaction completion, the reaction mixture was extracted to EtOAc and washed with DW. The organic layer was dried over sodium sulfate. The residue was purified by flash column chromatography (1:99 to 50:50; EtOAc/Hexane) to give a white solid (4.5 mg, 35 %). ^1^H NMR (600 MHz, CDCl_3_) δ 5.79 (dt, *J* = 15.6, 6.8 Hz, 1H), 5.64 (d, *J* = 9.1 Hz, 1H), 5.39 (dd, *J* = 15.4, 7.4 Hz, 1H), 5.28 (t, *J* = 6.7 Hz, 1H), 4.46 – 4.40 (m, 1H), 4.30 (dd, *J* = 11.6, 6.0 Hz, 1H), 4.04 (dd, *J* = 11.6, 4.0 Hz, 1H), 2.07 (d, *J* = 4.1 Hz, 6H), 2.05 – 2.00 (m, 2H), 1.98 (s, 3H), 1.35 (dd, *J* = 8.6, 7.4 Hz, 2H), 1.25 (s, 20H), 0.88 (t, *J* = 7.1 Hz, 3H). HRMS (ESI): [M+Na]^+^ m/z calcd. 448.30334 for C_24_H_43_NO_5_Na^+^, found 448.30347.

**^1^H NMR spectra**

**(2R,3S)-2-Acetamidooctadecane-1,3-diyl diacetate (1) – Triacetyl sphinganine**


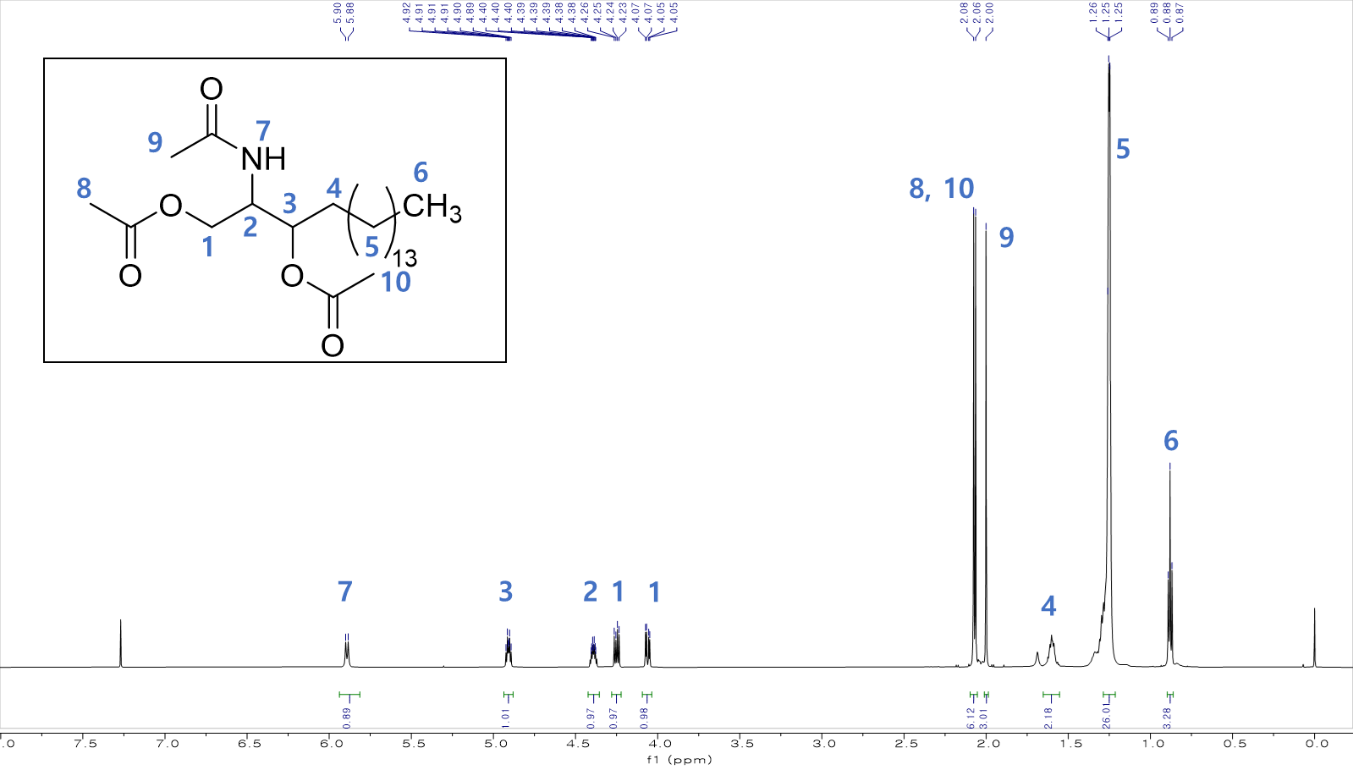


**(2R,3S,E)-2-Acetamidooctadec-4-ene-1,3-diyl diacetate (2) – Triacetyl sphingosine**


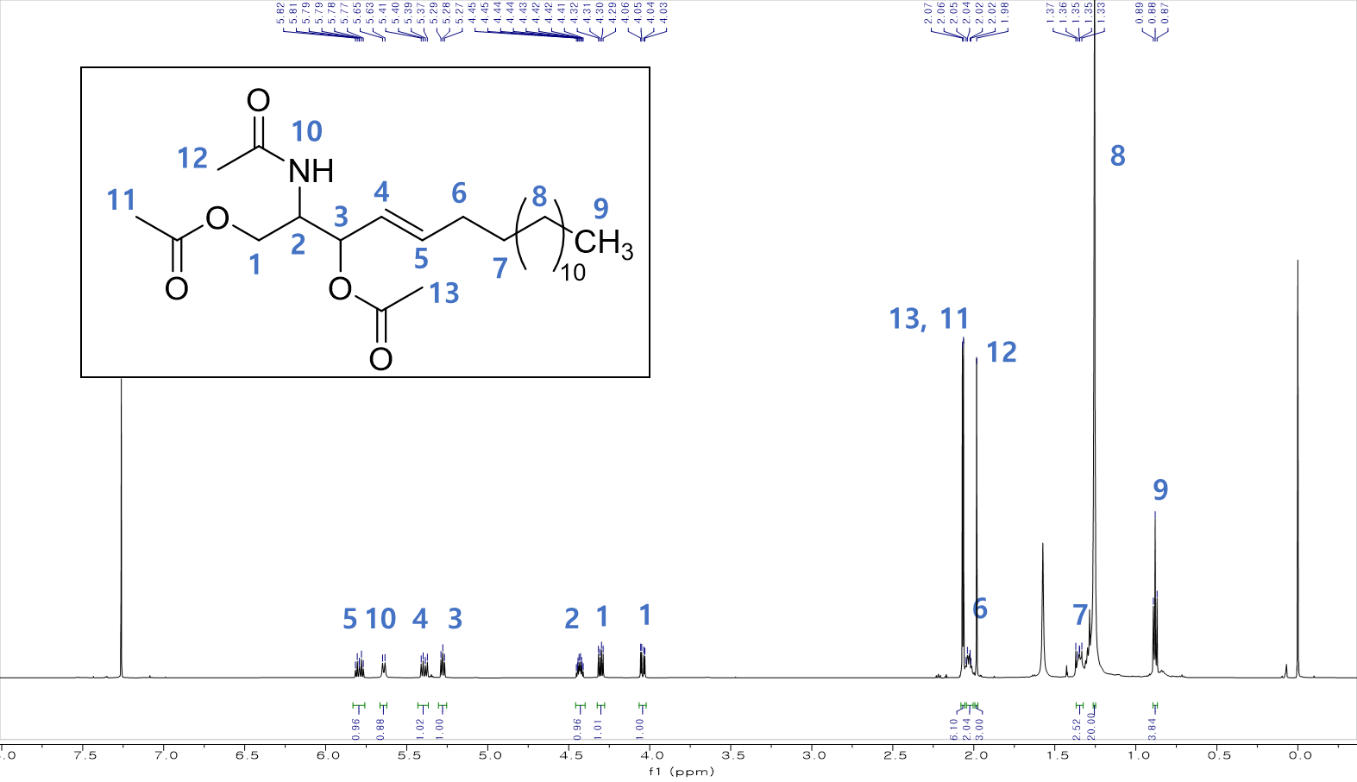


**Supplementary data 2**


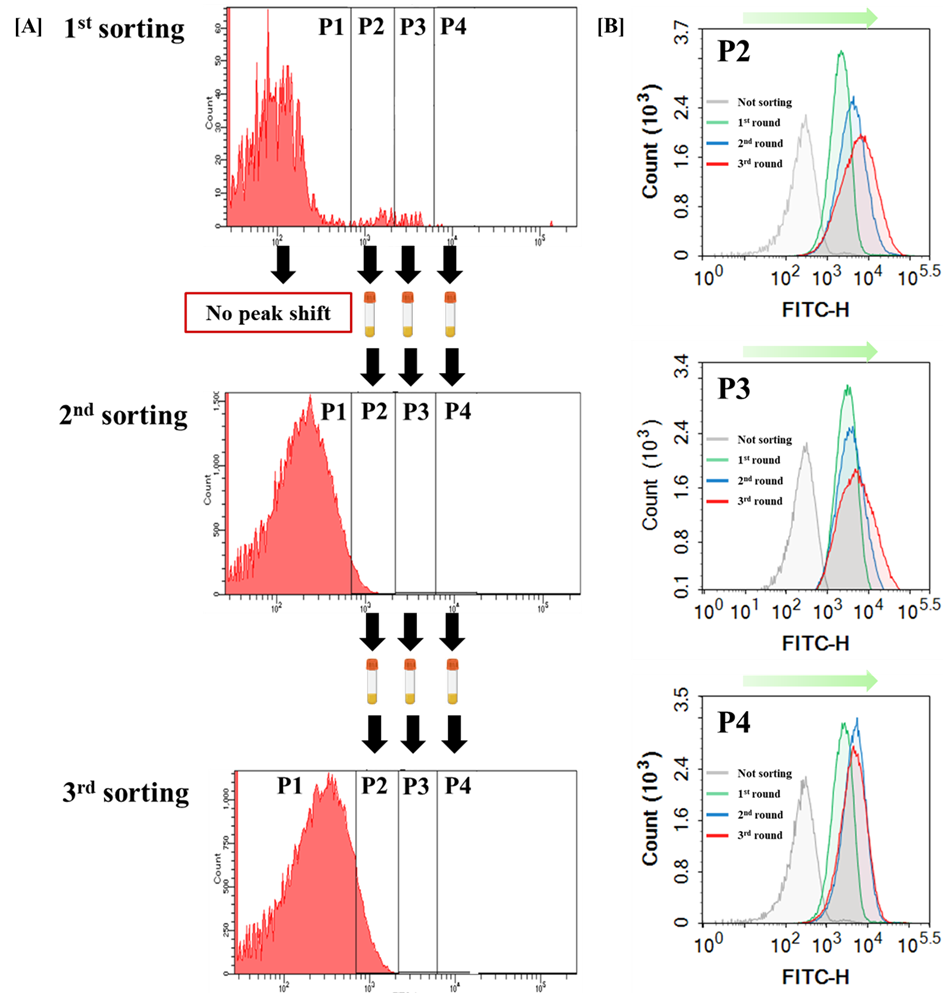


**FACS analysis of sorted cells across gate regions and sorting rounds after fluorescein sodium staining**. [A] FACS analysis of the mutant library stained with fluorescein sodium, showing gate regions P1 to P4, which were used to sort cells based on fluorescence intensity. Higher fluorescence intensity corresponds to P2 and P4 regions, where potential sphingoid base-overproducing mutants are enriched. [B] FACS analysis of fluorescence intensity progression for cells sorted from P2, P3, and P4 gates across multiple sorting rounds (gray: no sorting, green: 1 round, blue: 2 rounds, red: 3 rounds). Successive rounds of sorting resulted in a progressive shift toward higher fluorescence, demonstrating the enrichment of high-fluorescence cell populations indicative of elevated sphingoid base production.

**Supplementary data 3**


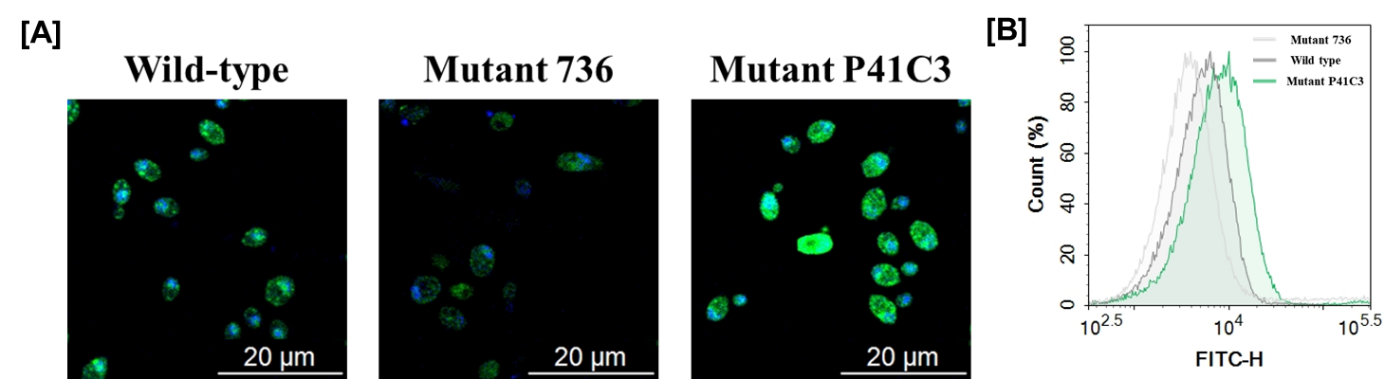


**Fluorescence intensity analysis using confocal imaging and FACS**. (A) Confocal imaging of *W. ciferrii* strains stained with fluorescein sodium (green, indicating sphingoid bases) and DAPI (blue, for nuclear staining). The wild-type strain, TAPS-overproducing mutant 736, and sphingosine-producing mutant P41C3 were analyzed. The P41C3 strain exhibits significantly higher fluorescein sodium staining intensity compared to the wild-type and mutant 736 strains, suggesting increased sphingosine accumulation. Scale bars: 20 μm. (B) FACS analysis comparing fluorescence intensity distributions among the wild-type strain (gray), mutant 736 (light gray), and sphingosine-producing mutant P41C3 (green). The P41C3 mutant shows a rightward shift in fluorescence intensity, confirming elevated sphingosine production *in vivo*.
